# The Roles of Gasdermin D in Coronavirus Infection and Evasion

**DOI:** 10.3389/fmicb.2021.784009

**Published:** 2021-11-26

**Authors:** Xiang Liu, Shihao Ding, Pinghuang Liu

**Affiliations:** Key Laboratory of Animal Epidemiology of the Ministry of Agriculture, College of Veterinary Medicine, China Agricultural University, Beijing, China

**Keywords:** gasdermin D, pyroptosis, inflammasomes, viruses, coronaviruses

## Abstract

Pyroptosis is lytic, programmed cell death and plays a critical role against microbial invasion, functioning as an innate immune effector mechanism. The pore-forming protein gasdermin D (GSDMD), a member of gasdermin family proteins, is a primary effector of pyroptosis. The cleavage of inflammasome-associated inflammatory caspases activates GSDMD to liberate the N-terminal effector domain from the C-terminal inhibitory domain and form pores in the cellular plasma membrane. Emerging evidence shows that the pore-forming activity of GSDMD beyond pyroptosis and modifies non-lytic cytosolic protein secretion in living cells and innate immunity. While the essential roles of GSDMD in bacterial infection and cancer have been widely investigated, the importance of GSDMD in virus infection, including coronaviruses, remains elusive. Here, we review the current literature regarding the activation and functions of GSDMD during virus infections. Last, we further discuss the roles of GSDMD and the therapeutic potential of targeting this GSDMD pore-forming activity in coronavirus diseases.

## Introduction

Pyroptosis, one of the types of programmed cell deaths (Pyroptosis, apoptosis, necroptosis), functions as the critical innate effector of host responses in microbial infection and cancer and has attracted considerable attention in recent years (Aglietti et al., 2016; Frank and Vince, 2019). As an important innate immune response, pyroptosis eliminates infected cells and restricts intracellular pathogens’ survival and proliferation (Bergsbaken et al., 2009; Schroder and Tschopp, 2010). Gasdermin D (GSDMD), a member of gasdermin family proteins, is widely expressed in various cell types, including epithelial cells and immune cells (Tamura et al., 2007; Orning et al., 2019). GSDMD has intensively been investigated in microbial infection and cancer since GSDMD was identified as the main executioner of pyroptosis in 2015 (Shi et al., 2015; Orning et al., 2019). GSDMD N-terminus is released from the autoinhibitory form after cleavage and undergoes oligomerization to form membrane pores that drive leakage and a programmed lytic cell death, pyroptosis (Ding et al., 2016). The plasma membrane pores formed by GSDMD N-terminus oligomerization also serve as conduits for the transport of inflammatory cytokines across intact membrane lipid bilayers and contribute to the host inflammatory responses (Evavold et al., 2018). Therefore, GSDMD plays a crucial role in the host response to microbial infection, including viruses.

Coronaviruses are a large family of enveloped viruses with positive sense, non-segmented, single-stranded RNA genomes. Coronaviruses infect many hosts, from mammals to birds, and cause serious public health threats and economic impact (Cui et al., 2019). The recently emerging severe acute respiratory syndrome coronavirus 2 (SARS-CoV-2), a member of human betacoronavirus, is the causative agent of coronavirus disease 2019 (COVID-19) which has become a global pandemic and resulted in more than 4 million human deaths (WHO, 2021). The pathogenesis of coronaviruses is related to both virus replication and the inflammatory responses elicited by virus infection. However, the underlying pathogenesis of coronavirus-associated diseases remains elusive, though there have been intensive coronavirus studies since the breakout of COVID-19. In this review, we discuss recent studies about the functions of GDSMD in viral infectious diseases with a special focus on how GSDMD modifies coronavirus infection.

## Overview of Gasdermin D Structure and Pore-Forming Activity

**FIGURE 1:**
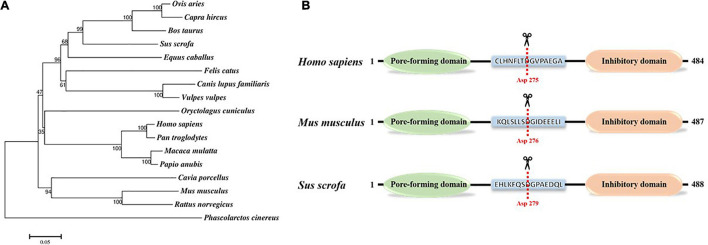
Gasdermin D protein. **(A)** Phylogenetic tree of gasdermin D protein. The numbers on the nodes indicate the percentage of times the species grouped in the bootstrap tree. Amino acid sequence data of GSDMD in different mammals were obtained from UniProt (www.uniprot.org). The Phylogenetic tree was generated via MEGA 7 software using the neighbor-joining method with bootstrap values obtained from 1,000 replications. The scale bar indicates the estimated evolutionary distance. **(B)** Schematic diagram of Homo sapiens, Mus musculus, and Sus scrofa GSDMD along with key residue. The red dotted line marks the catalytic center of GSDMD that cleaved by cysteine protease at D275 in Homo sapiens and D276 in Mus musculus, and we predicted that the cleavage site of Sus scrofa GSDMD was D279.

GSDMD is a member of the gasdermin family that consists of GSDMA, GSDMB, GSDMC, GSDMD, GSDME, and Pejvakin in humans (Ruan, 2019). The homology of the gasdermin family members is about 45%. All the gasdermins, except DFNB59, adopt a similar structure that is composed of a pore-forming domain (PFD) and repressing domain (RD) (Shi et al., 2015, 2017). However, the gasdermin proteins have a different intermediate sequence, consisting of nine conserved regions, all of whose members encode leucine-rich proteins (Qiu et al., 2017). Except for DFNB59, which lacks the key domain responsible for the pore-forming activity, most members of the gasdermin family can form pores in lipid membranes (Shi et al., 2015). Among them, GSDMD is the most thoroughly studied protein in the gasdermin family. GSDMD is mainly expressed in the skin, stomach, and esophagus (Tamura et al., 2007; Feng et al., 2018), which are the initial sites of pathogen invasion, suggesting that GSDMD may play an important role in host defense. GSDMD consists of 484 amino acids with a total length of about 53 kDa in humans or pigs, and the N-terminal pore-forming domain and the C-terminal repressing domain are connected by a ligand containing dozens of amino acids (Saeki and Sasaki, 2012; Figure 1). Based on the amino acid sequence data obtained from UniPort,^1^ the GSDMD protein phylogenetic tree of different species (GSDMD is not detected in chicken, duck, goose, and other avian animals) was constructed via MEGA 7 software using the neighbor-joining method. The bootstrap scores observed for all the nodes are indicated, and the length of “branch” is closely related to evolutionary distance. As shown in Figure 1A, the GSDMD of different species are clustered together separately and differentiated into 3 discernible major “branches,” indicating the common origin and evolutionary relationship of GSDMD among different species. The GSDMD of Phascolarctos cinereus and other mammals exhibited a distant relationship. However, the GSDMD of other mammals is clustered together into a single branch. Its root node is the GSDMD of Phascolarctos cinereus, suggesting that the GSDMD of Phascolarctos cinereus may be the common ancestor of the GSDMD of other mammals.

What is more, GSDMD protein contains characteristic gasdermin domain, and activated caspase-1 and caspase-11 can cleave and activate GSDMD at Asp276 in mice or Asp275 in humans to promote its activation (Liu et al., 2016). Based on the cleavage sites of mice and humans, we predicted that the cleavage site of pig GSDMD was D279 (Figure 1B). Sequence homology suggests that members of the gasdermin family (except for DFNB59) share similar 3D structures, and the crystal structure of GSDMD is highly similar to that of GSDMA3. The N-terminal pore-forming domain of GSDMD has an extended twisted β-sheet core structure, and the C-terminal repressing domain is a compact α-helical globular fold. Ligand with caspase cutting site binds the two domain ends together through a pocket-like structure (Ding et al., 2016). GSDMD-C-terminal inhibits GSDMD-N-terminal activity and binds to autoprocessing p10 fragment of caspase-1/4 to positively promote GSDMD cleavage and further induce pyroptosis (Liu et al., 2018, 2020). Many studies have shown that this ligand of GSDMD is cleaved by activated cysteine protease caspase-1, -8, -11 at residue D275 in human GSDMD, then, the α4 helix is released from the pocket structure, and GSDMD is cleaved into a 30 kDa lipophilic GSDMD-N-terminal domain and a 23 kDa hydrophilic GSDMD-C-terminal domain (Sborgi et al., 2016). Once the self-inhibition structure is released, the GSDMD-N-terminal domain could translocate to cytomembrane to bind acidic lipids, such as cardiolipin (CL) phosphatidylserine (PS) and phosphatidylinositol phosphates (PIPs) (Ding et al., 2016; Liu et al., 2016). Pore-forming activity of the GSDMD-N-terminal domain is activated, and the oligomerization of approximately 16 PFD monomers form a hole in the cell membrane with a diameter of 10–15 nm, which is wide enough to allow the free flow of small-sized proteins such as mature IL-1β and IL-18 across the membrane (Chen et al., 2016). When there are too many holes in the membrane, the osmotic pressure inside and outside the membrane will be unbalanced, which leads to membrane disruption and eventually causing apoptosis (Ding et al., 2016; de Vasconcelos et al., 2019).

GSDMD permits inflammatory cytokines passing across intact lipid bilayers in hyperactive living cells and plays critical roles in the non-lytic IL-1β/IL-18 cytokine release (Evavold et al., 2018). In addition, the C-terminal of GSDMD can return to the N-terminal of GSDMD and repress its activity (Liu et al., 2016). Some researchers also demonstrated that the activated GSDMD-N-terminal domain recombinant protein could disrupt cytomembrane from the inside of eukaryotic cells, whereas GSDMD-N-terminal domain recombinant protein that added directly to cell supernatants could not induce pyroptosis, which is consistent with the fact that membrane lipids and phosphoinositide are only distributed on the inner side of cell membranes (Ding et al., 2016).

Efficient cleavage of GSDMD by cysteine protease is critical for N-terminal release from the inhibitory grip of the C-terminus. It was shown by Shi et al. (2015) and Wang et al. (2020) that mutation at the residue of D275 results in non-cleavable and non-functional GSDMD. Mutation at the residue of E15 results in no spontaneous pyroptosis-inducing activity, and mutation of two amino acids at I104 or L192 in N-terminal resulted in decreased induction of pyroptosis (Sborgi et al., 2016; Kuang et al., 2017). Additional attempts to understand the key position of the C-terminal domain of GSDMD come from Ding et al. (2016) that mutation of L290, Y373, and A377 lead to autoactivation of GSDMD. The fact that the N-terminal of GSDMD is the only effector to form a pore in the membrane was demonstrated by Sborgi et al. (2016) using atomic force microscopy, and they observed that the activated GSDMD-N-terminal domain binds to the artificial biomembrane directionally and polymerize to form a hollow circular oligomer, which then form pores and trigger cell pyroptosis. This conclusion has also been further confirmed in other groups by negative staining electron microscopy (Aglietti et al., 2016; Ding et al., 2016).

## The Gasdermin D-Mediated Pyroptosis

Cookson and Brennan (2001) discovered in 2001 that caspase-1 is activated when macrophages are infected with salmonella and then leads to membrane lysis and recruitment of nearby macrophages. This programmed cell death process was defined as "pyroptosis" due to its inflammatory response and was classified as a new form of cell death that is distinct from apoptosis, often accompanied by cell swelling, the release of inflammatory cytokines, and disruption of the cell membrane (Cookson and Brennan, 2001; Jorgensen and Miao, 2015). Pyroptosis is an important protective host defense measure of innate immunity, which plays an important part in response to pathogen infection. It was deemed an inflammatory programmed cell death process dependent on caspase-1 for a long time (He et al., 2015). With further research development, some researchers have found that GSDMD is a common cleavage substrate of caspase-1 and caspase-11/4/5, and the cleavage process is essential for pyroptosis occurrence (Shi et al., 2017). Therefore, there is increasing interest in pursuing GSDMD as a new therapeutic target for anti-infection treatment.

### Canonical Pathway

The process that pattern recognition receptors (PRRs) recognize PAMP (pathogen-associated molecular pattern) of pathogenic microorganisms is the premise and basis of innate immunity. Several PRRs have been shown to form inflammasome with apoptosis-associated speck-like protein (ASC) and pro-caspase-1 (Malik and Kanneganti, 2017). After assembly, inflammasomes could recognize PAMPs, such as bacteria, fungi, viruses, and DAMPs (damage-associated molecular pattern), such as ATP and cholesterol, and further, promote the secretion of IL-1β and IL-18 by immune cells, and eventually lead to pyroptosis (Man et al., 2017). In the canonical pathway, PRRs recruit ASC and pro-caspase-1 to form inflammasome after recognizing PAMPs or DAMPs. The pro-caspase-1 is subsequently cleaved into its bioactive form-caspase-1. On the one hand, activated caspase-1 can promote the cleavage of pro-IL-1β and pro-IL-18.

On the other hand, GSDMD is cleaved by caspase-1 within the linker between the GSDMD N-terminal domain and GSDMD C-terminal domain to break autoinhibitory interaction. The GSDMD N-terminal domain binds to PIPs on the cell membrane and oligomerizes to form a pore diameter of 10–15 nm (Mulvihill et al., 2018). Mature IL-1β and IL-18 are released across the membrane through the GSDMD-N pores (Chen et al., 2016). When there are too many pores in the cell membrane, the intracellular and extracellular osmotic pressure are unbalanced, resulting in cell swelling and membrane lysis. The immune cells are recruited and trigger the inflammatory response, leading to pyroptosis (Shi et al., 2017).

### Non-canonical Pathway

**FIGURE 2:**
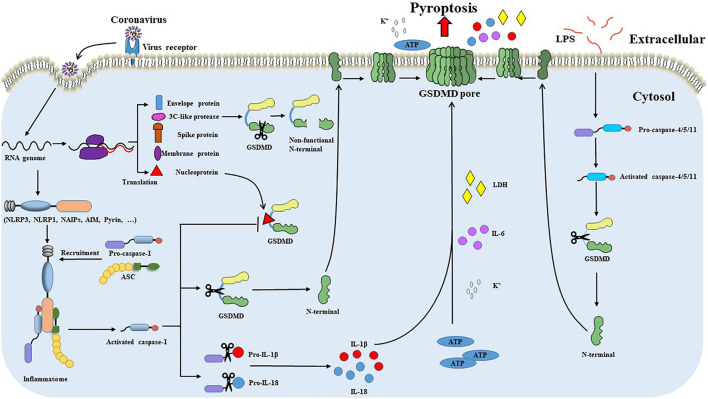
GSDMD-mediated pyroptosis during coronavirus infections. After recognizing and binding to the cell surface receptor, the coronavirus enters the cell through internalization and carries out transcription and translation processes inside cells. Intracellular viral RNA initiates signaling through cytosolic pattern recognition receptors, such as NLRP1, NLRP3, and AIM2. PRRs recognize viral RNA and recruit ASC and pro-caspase-1 to form inflammasome and promote caspase-1 activation. Activated caspase-1 promotes the cleavage of pro-IL-1β and pro-IL-18. Meanwhile, GSDMD is cleaved by caspase-1 within the linker between the GSDMD N-terminal and GSDMD C-terminal domains. The GSDMD N-terminal domain binds to the cell membrane and oligomerizes to form a pore. Pore formation leads to the influx of water molecules and the secretion of IL-1β and IL-18, which eventually leads to cell swelling and subsequent pyroptosis. The nucleoprotein of some coronavirus, such as SARS-CoV-2, can effectively block the caspase-1 mediated cleavage of GSDMD by binding the GSDMD linker region. Furthermore, 3C-like protease of coronavirus incorrectly cleaves GSDMD and produces a non-functional N-terminal. Coronaviruses escape from GSDMD-mediated pyroptosis through the pathways mentioned above. In addition to the canonical pyroptosis pathway, caspase-4/5/11 can also induce pyroptosis through the non-canonical pathway. LPS binds to procaspase-4/5/11 and promotes the activation of caspase-4/5/11. Activated caspase-4/5/11 can also cleave the GSDMD linker to generate the pore-forming GSDMD-N terminal, causing potassium (K^+^) outflow and NLRP3 inflammasome activation leading to pyroptosis.

In addition to caspase-1, caspase-11 in mouse macrophage and human caspase-4/5 can also induce pyroptosis (Frank and Vince, 2019). Caspase-11 and caspase-4/5 bind specifically to LPS, and activated caspase-11 cleaves GSDMD to release the N-terminal effector domain of GSDMD, and the released N-terminal effector domain forms pores in cell membranes to induce potassium (K^+^) outflow and NLRP3 inflammasome activation. Activated NLRP3 inflammasome further cleaves caspase-1 to release (Kayagaki et al., 2011; Rühl and Broz, 2015). In addition, caspase-11 can also activate the pannexin-1 pathway to release ATP and then promote the opening of the membrane channel P2X 7, which eventually leads to the occurrence of pyroptosis (Moreira-Souza et al., 2017). Although the inflammasome participates in non-canonical pathways, it does not directly participate in the process of pyroptosis. Inflammasome cleaves GSDMD through inflammatory cysteine protease, making GSDMD have pore-forming activity and further causing pyroptosis. This regulatory approach suggests that the canonical and non-canonical pathways can be transformed into each other and interact through NLRP3 inflammasome (Figure 2).

## The Gasdermin D Activity in the Inflammatory Response

Pyroptosis is a kind of programmed cell death that has been gradually defined in recent years (Frank and Vince, 2019). Unlike apoptosis, pyroptosis is closely related to inflammatory responses and releases inflammatory mediators simultaneously with cell death. As an important part of innate immunity, on the one hand, pyroptosis plays a role in immune protection through inflammatory response to eliminate intracellular risk factors. On the other hand, the over-activation of pyroptosis can also lead to excessive inflammatory response and cause damage to the host.

In the canonical pyroptosis pathway, inflammasome assembly promotes the cleavage of caspase-1 and the secretion of inflammatory cytokines IL-1β and IL-18 to induce the inflammatory response. In 2015, Kayagaki et al. (2015) found that knockout of GSDMD in mice resulted in decreased secretion of Il-1β and impaired LDH release. As we all know, IL-1β is a cytosolic protein that cannot be secreted through the classical ER-Golgi secretion pathway, for it lacks targeting sequences (Rubartelli et al., 1990; Gu et al., 1997; Kayagaki et al., 2015). GSDMD knockout BMDMs showed that mature IL-1β is abundant and stranded in the cytoplasm and fails to secrete across the cytomembrane when stimulated by other stimuli (He et al., 2015; Kayagaki et al., 2015; Shi et al., 2015). In addition, Kanneganti et al. (2018) and Xiao et al. (2018) proved that the upregulated IL-1β induced by familial Mediterranean fever (FMF) and neonatal-onset multisystem inflammatory disease was reduced in *Gsdmd*^–/–^ mice. It has long been believed that the secretion of inflammatory cytokine in the non-canonical pyroptosis pathway depends on the cleavage of GSDMD, which forms pores in cytomembrane to allow K^+^ efflux to active NLRP3 inflammasome and then promotes the secretion of IL-1β (Kayagaki et al., 2011; Rühl and Broz, 2015). It is consistent with the above conclusions that *Gsdmd*^–/–^ macrophages can still activate caspase-11 following LPS transfection but cannot cleave pro-caspase-1 or pro-IL-1β/pro-IL-18 and induce pyroptosis (Shi et al., 2015; Ramirez et al., 2018). In agreement, in the case of intrinsic and extrinsic induction of apoptosis in macrophage, NLRP3 inflammasome activation mediated by GSDMD cleavage has also been proved in the context of caspase-8 activation, and this process promotes the maturation and secretion of IL-1β (Chauhan et al., 2018; Orning et al., 2018; Sarhan et al., 2018). It was further demonstrated by Xu et al. (2018) that *Gsdmd*^–/–^ mice showed decreased severity of inflammation and resistance to steatosis induced by methionine-choline deficient feeding. Pyroptosis release intracellular contents such as inflammatory cytokines that act on neighboring cells to induce the inflammatory response. In addition to IL-18 and IL-1β, IL-1α is also released in the process of pyroptosis. It was reported by Kayagaki et al. (2011) that caspase-1/-11 induced pyroptosis controls the release of IL-1α on several inflammasome triggers. Therefore, GSDMD is a key factor of inflammatory cytokine release *in vitro* and *in vivo*.

Recent studies have revealed that GSDMD can also promote the occurrence of inflammatory responses through multiple mechanisms. GSDMD can induce the release of IL-1β through the non-pyroptosis approach and thus play a similar role to pyroptosis. For example, in neutrophil granulocytes, GSDMD N-terminal does not migrate to cytomembrane to form pores but migrate to azurophilic granules and autophagosomes after cleavage and activation of GSDMD, which causes pores formation in cytomembrane through autophagy and promotes the release of IL-1β (Karmakar et al., 2020). In addition, activation of caspase-8 in intestinal epithelial cells also promotes the non-pyroptotic role of GSDMD and causes the release of IL-1β containing small extracellular vesicles through exocytosis (Bulek et al., 2020). These inflammatory mediators may act as exogenous risk factors, in turn, to promote the secretion of IL-1β. These results suggest that GSDMD is involved in the pathogenesis of intestinal inflammation. GSDMD in macrophages may have both pro-inflammatory and anti-inflammatory effects. In the colitis model, mice GSDMD can relieve intestinal inflammatory symptoms by inhibiting the cGAS-STING signaling pathway, and this process has nothing to do with intestinal flora and is mainly induced by K^+^ outflow caused by GSDMD pore formation in the cytomembrane (Ma et al., 2020).

Studies have shown that pyroptosis is involved in the pathogenesis process of many diseases, such as infectious diseases, autoimmune diseases, atherosclerosis, and tumors (Man et al., 2017; McKenzie et al., 2018; Fan et al., 2019). It was shown by Xiao et al. (2018) that *Gsdmd*^–/–^ mice were relieved from a skin lesion, enlarged spleen, and growth restriction induced by neonatal-onset multisystem inflammatory disease. Neutrophil infiltration was further reduced in the liver, subcutaneous tissue, and spleen (Xiao et al., 2018). In recent years, Kanneganti et al. (2018) proved that FMF (a kind of systemic autoinflammatory diseases) model mouse by Clostridium difficile induces pyroptosis and promotes the secretion of IL-1β, and further *in vivo* experiment showed that the expression level of IL-1β was reduced and the organ-specific inflammatory injury, such as hepatitis, glomerulonephritis, and colitis was also relieved when GSDMD was knocked out. These studies suggest that GSDMD is also involved in the pathogenesis of systemic autoinflammatory diseases and is expected to become a new target for treating systemic autoinflammatory diseases.

## The Modification of the Type I IFN Response by Gasdermin D Activity

Type I IFN is the initial host innate cytokine in response to infection and plays a critical role in host defense against virus infection. Type I IFNs are induced by the stimulation of receptors known as PRRs, including Toll-like receptors (TLRs), RNA sensor DDX58 and MDA5, DNA sensor cGAS, and Z-DNA combined protein-1 (ZBP-1) (Collins and Mossman, 2014). The antiviral activity of IFNs is mediated by inducing a wide range of ISG genes, which promotes an antiviral state in bystander cells and restricts viral replication. In addition to its antiviral activity, type I IFN signaling promotes and regulates immune and inflammatory responses and controls several types of cell death. GSDMD is the primary effector of canonical pyroptosis and is integrated with the host inflammatory responses. However, the study of GSDMD in modifying type I IFNs is sporadic. The DNA sensor cGAS plays an essential role in inducing type I IFN in response to microbial DNA stimulation (Schneider et al., 2014). A recent study shows that GSDMD pore-forming activity activated by AIM2 inflammasome leads to cytosolic K^+^ efflux and the reduction of intracellular K^+^, which dampens cGAS-dependent type I IFN elicitation (Banerjee et al., 2018), which is consistent with another bacterial study that spontaneous combustion caused by the cytoplasmatic Rickettsia parkeri pathogen inhibits type I IFN production and benefits Rickettsia parkeri infection (Burke et al., 2020). Whether GSDMD manipulates the production of type I IFNs in the scenario of virus infection is still unclear.

## The Role of Gasdermin D Activity in Virus Infection

Pyroptosis is well established as an innate host defense mechanism against intracellular pathogens and facilitates host defense against pathogenic microorganisms by eliminating infected cells and thereby curtailing the survival and proliferation of intracellular pathogens. Recent studies have demonstrated that various virus infections result in pyroptosis. During human adenoviruses (HAdV) infection, AIM2 inflammasome can activate caspase-1 and facilitate GSDMD cleavage in monocyte-derived DCs, resulting in pyroptosis (Eichholz et al., 2016). Dengue virus infection causes caspase 1-mediated pyroptosis (Tan and Chu, 2013). It is not surprising that GSDMD as a primary effector of pyroptosis involves viral infection pathogenesis. While the functions and importance of GSDMD as a regulator of inflammasome activities in response to cytosolic bacterial infection or LPS stimulation have been extensively investigated, the roles of GSDMD during virus infection remain unclear. As the main pyroptosis executioner, GSDMD helps release inflammatory cytokines, including bioactive IL-1β and IL-18, which contribute to a series of antiviral immunity and inflammation. GSDMD knockout in the intestine *in vivo* significantly enhances the susceptibility of mice to rotavirus infection, indicating a protective role of GSDMD against rotavirus infection. However, the underlying mechanisms of GSDMD viral inhibition are unclear (Zhu et al., 2017). GSDMD pore-forming activity can be a double-edged sword with both beneficial and detrimental effects for the host. In some circumstances, GSDMD pore-forming activity also has a detrimental function. Norovirus infection of STAT1-deficient mice can activate canonical NLRP3 inflammasome and lead to the maturation of IL-1β and GSDMD-dependent pyroptosis, which contributes to immunopathology during gastrointestinal norovirus infection (Dubois et al., 2019).

Similarly, He et al. (2020) identified that a caspase-1-GSDMD mediated pyroptotic cell death in neural progenitor cells induced by Zika virus infection contributes to the Zika virus-induced brain atrophy. In addition, viruses have also evolved the specific mechanism to inhibit pyroptosis by directly targeting GSDMD. Enterovirus 71 protease 3C can unfunctionally cleave GSDMD at Q193-G194 in the N-terminus to inhibit GSDMD activity, which is used to escape the defense of host antiviral immunity (Lei et al., 2017). By and large, the interaction effect of GSDMD activity and virus infection is complicated, which remains to be further elucidated.

## The Functions of Gasdermin D Activity in Coronavirus Infection

Based on their genetic and serologic properties, coronaviruses can be classified into four genera (alpha, beta, gamma, and delta) (Cui et al., 2019). Coronaviruses have a broader host spectrum and pose serious health threats to humans and animals. Coronaviruses generally target the respiratory and gastrointestinal systems. The disease severity in patients after coronavirus infection is due to the viral infection and the host inflammatory responses. GSDMD is widely expressed in skin and mucosal epithelial immune cells (Tamura et al., 2007; Orning et al., 2019). The highly pathogenic human coronaviruses (SARS-CoV-2, SARS-CoV) replicate in the lower respiratory tract and cause acute respiratory distress syndrome (ARDS), severe pneumonia-like symptoms (Cui et al., 2019; Vora et al., 2021). The SARS-CoV-2 infection causes inflammasome activation and pyroptosis in blood monocytes, and cleaved GSDMD was also detected in lung tissue sections and bronchoalveolar lavage fluid (BALF) patients with COVID-19 (Vora et al., 2021). Signs of pyroptosis (IL-1 family cytokines, LDH) in the plasma and a higher GSDMD expression are linked to the increased risk of severe COVID-19 disease (Vora et al., 2021; Zhang et al., 2021). These all suggest a critical role for GSDMD in COVID-19 pathogenesis. In the small intestine, GSDMD cluster genes were widely expressed in the small intestine villi and gradually decreased toward the distal small intestine. Swine enteric coronaviruses (PEDV, TGEV, PDCoV), importantly economic impact pathogens of the porcine industry, mainly infect small intestinal epithelia and cause high morbidity and mortality in neonatal pigs. TGEV infection in the intestine epithelia induced the production of pro-IL-1β and cleavage of GSDMD (Wei et al., 2021). GSDMD-dependent release of IL-1β is detected in cultured colonic explants and related to the pathogenesis of intestinal inflammation in a mouse model (Bulek et al., 2020). The plasma membrane pore formed by GSDMD plays an important part in inflammatory cytokine non-lytic secretion from living epithelia (Xia et al., 2021). Therefore, GSDMD potentially plays an important role in coronavirus-associated inflammation (Figure 2).

Cytopathic viruses such as SARS-CoV-2 and TGEV result in the death and injury of virus-infected cells as part of the viral replication cycle. Pyroptosis is a highly inflammatory form of programmed cell death and is one of the consequences of cytopathic viruses. The mechanistic details of the pyroptosis induced by coronaviruses and the functional consequences of pyroptosis have not been well elucidated. A major unanswered question is whether GSDMD serves protective or detrimental functions for coronavirus infection. A recent study shows that coronavirus-induced pyroptosis is primarily mediated by the GSDMD/caspase-1 canonical pathway instead of the GSDME/caspase-3 pathway (Zheng et al., 2020). GSDMD knockout increased the murine coronavirus mouse hepatitis virus (MHV) replication in bone marrow-derived macrophages (BMDMs) (Zheng et al., 2020), which is consistent with another study that inhibition of NLRP3 enhanced the replication of TGEV in intestinal epithelial cells (Wei et al., 2021). These all indicate that GSDMD undermines coronavirus replication, consistent with rotavirus results in which GSDMD knockout potently enhances rotavirus infection (Zhu et al., 2017).

Meanwhile, coronaviruses have evolved multiple strategies to evade the GSDMD-mediated pyroptosis. Recently Ma et al. (2021) showed that SARS-CoV-2 nucleoprotein effectively blocks the cleavage of GSDMD by binding the GSDMD linker region and suppressing GSDMD-mediated pyroptosis and cytokine release. We also found that the 3C-like protease of TGEV, like Enterovirus 71 protease 3C, incorrectly cleaves GSDMD and produces a non-functional N-terminal (unpublished data) (Figure 2). These limited studies indicate that GSDMD pore activity is detrimental to coronavirus replication. Thus, it is urgent to unravel the role of GSDMD in coronavirus infection fully.

## Conclusion and Perspectives

Coronaviruses constitute a serious impact on humans and animals. Most coronaviruses generally target respiratory and gastrointestinal epithelia *in vivo*. GSDMD, a main executioner of pyroptosis, is highly expressed in the epithelial cells and immune antigen-presenting cells. Recent studies have uncovered the molecular and biochemical mechanisms governing activation and functions of GSDMD in microbial infection and cancer. However, the functional relevance of GSDMD has not been well investigated in the context of coronavirus infection. Whether the GSDMD pore-forming activity is protective in the context of coronavirus-associated diseases awaits further *in vivo* or *in vitro* study. The degree and timing of GSDMD pore-forming activity during coronavirus infection may be critical. A better understanding of the mechanisms and functions of GSDMD during coronavirus infection may provide therapeutic options for coronavirus infection by targeting either to harness the beneficial effects or to block the harmful effects of GSDMD pore-forming activity.

## Author Contributions

PL, XL, and SD contributed to the content discussion, wrote the article, reviewed, and edited the manuscript before submission. All authors contributed to the article and approved the submitted version.

## Conflict of Interest

The authors declare that the research was conducted in the absence of any commercial or financial relationships that could be construed as a potential conflict of interest.

## Publisher’s Note

All claims expressed in this article are solely those of the authors and do not necessarily represent those of their affiliated organizations, or those of the publisher, the editors and the reviewers. Any product that may be evaluated in this article, or claim that may be made by its manufacturer, is not guaranteed or endorsed by the publisher.

## Footnotes

1
www.uniprot.org
